# Gene Therapy Strategies for HIV/AIDS: Preclinical Modeling in Humanized Mice

**DOI:** 10.3390/v5123119

**Published:** 2013-12-12

**Authors:** Michael S. Bennett, Ramesh Akkina

**Affiliations:** Department of Microbiology, Immunology and Pathology, Colorado State University, 1619 Campus delivery, Fort Collins, CO 80523, USA; E-Mail: Michael.Bennett@colostate.edu (M.S.B.)

**Keywords:** gene therapy for HIV/AIDS, humanized mice for HIV gene therapy, stem cell-based gene therapy, anti-HIV RNA-based therapies, new generation humanized mice

## Abstract

In the absence of an effective vaccine and lack of a complete cure, gene therapy approaches to control HIV infection offer feasible alternatives. Due to the chronic nature of infection, a wide window of opportunity exists to gene modify the HIV susceptible cells that continuously arise from the bone marrow source. To evaluate promising gene therapy approaches that employ various anti-HIV therapeutic molecules, an ideal animal model is necessary to generate important efficacy and preclinical data. In this regard, the humanized mouse models that harbor human hematopoietic cells susceptible to HIV infection provide a suitable *in vivo* system. This review summarizes the currently used humanized mouse models and different anti-HIV molecules utilized for conferring HIV resistance. Humanized mouse models are compared for their utility in this context and provide perspectives for new directions.

## 1. Introduction

The advent of combinatorial antiretroviral therapy proved to be highly effective in controlling HIV disease progression to full blown AIDS and thus in dramatically decreasing the mortality rates where treatment is available [1]. However, major current issues are systemic drug toxicity and generation of drug resistant viral mutants during prolonged therapy. In addition, viral latency remains an intractable problem [2,3]. Therefore, alternative innovative approaches are continually being pursued to provide an effective cure. Among these are immune-augmentation protocols to reinvigorate the immune system [4] and gene therapy strategies to render HIV susceptible cells impervious to virus infection at the entry level and/or interfere with intracellular virus replication [5,6]. These approaches show considerable promise due to the inherent nature of HIV infection and disease progression. First, unlike acute viral diseases wherein pathogenesis is rapidly occurring in a span of few days, HIV is a chronic disease, thus providing a wide window of opportunity to interfere with the slow disease progression. Second, the viral susceptible cells are of hematopoietic origin, which are generated on a continued basis from the bone marrow source wherein the precursor cells reside. Thus, therapies can be effectively directed to the newly generated hematopoietic cells, or alternatively the precursor stem cells themselves can be genetically altered such that their progeny can be made virus resistant.

For the success of HIV gene therapy strategies, a number of important criteria need to be met: (1) An effective gene therapeutic construct should be able to permanently disable a critically needed host factor for viral infection and/or potently inhibit virally-encoded messages and/or proteins. (2) Appropriate gene delivery vehicles with minimal toxicity such as gene transducing viral vectors are needed that have high efficiency of gene transduction into HIV target cells or their progenitors. (3) The anti-HIV genes should have long term efficacy and should not promote generation of viral escape mutants. (4) They should not display adverse effects on lineage specific differentiation and immunological function. (5) Finally, promising anti-HIV gene therapeutic constructs need to be evaluated in a suitable *in vivo* system to derive critical preclinical data necessary for subsequent human clinical trials. This review is mainly focused on currently available humanized mouse models and their utility in testing a variety of anti-HIV gene constructs.

## 2. An Ideal *in Vivo* Animal Model for HIV Gene Therapy

**Figure 1:**
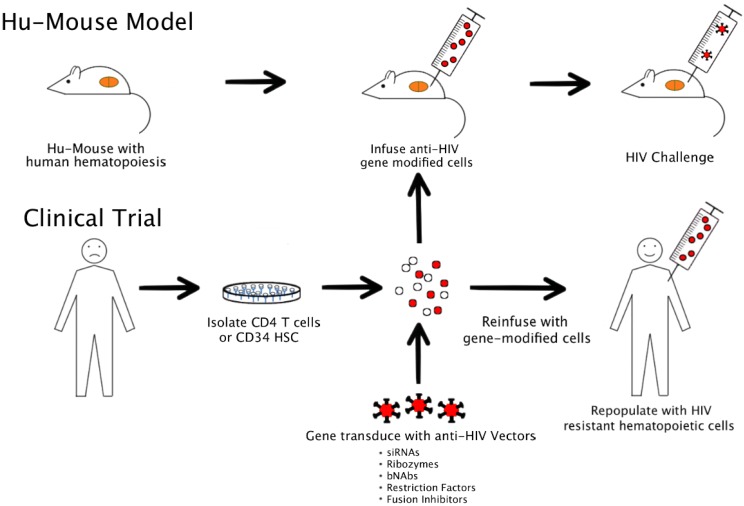
Modeling HIV gene therapy in humanized mice and clinical application.

HIV is a human virus causing severe disease in its natural host. While chimpanzees can be infected with HIV, they rarely show severe disease. In comparative studies, non-human primate (NHP) macaque models employing related simian immunodeficiency virus (SIV) and chimeric viruses such as simian-human immunodeficiency viruses (SHIVs) have yielded important data [5]. However, their utility is somewhat limited to derive full-fledged relevant data on HIV. In this regard, humanized mice transplanted with HIV susceptible human cells currently are becoming indispensable for testing various anti-HIV constructs [7] (Figure 1). While a variety of humanized mice are currently available, an ideal model should satisfy the following criteria. (1) They should harbor HIV susceptible cells long term and permit chronic HIV infection and helper CD4 T cell loss. (2) Ideally they should continuously generate the full spectrum of HIV susceptible cells, namely CD4 T cells, macrophages and dendritic cells which are primary viral targets. (3) They should permit HIV viral latency as seen in a typical HIV patient. (4) Finally they should generate human immune responses such that immune-restoration by gene therapy strategies can be effectively evaluated.

## 3. Immunodeficient Strains Used to Generate Humanized Mice

Various humanized mouse models have been used to test gene therapy strategies since the concept of intracellular immunization for HIV was conceived [7,8]. A common denominator has been the utilization of immunodeficient mice which do not reject xenografts for human cell reconstitution. Among the early immunocompromized mice is the SCID mouse which lacks T and B cells which permitted creation of hu-PBL-SCID and SCID-hu mouse models [9,10,11,12]. Later improvements led to generation of NOD-SCID mice with lower levels of NK cells and innate immunity, permitting improved levels of human cell engraftments [13]. A subsequent innovation was the targeted inactivation of the murine IL-2 receptor common gamma chain (IL2-Rcγ) gene, thus nullifying the actions of native mouse cytokines IL-2, IL-4, IL-7, IL-9, IL- 15 and IL-21 [13,14]. This trait, when bred into mice harboring SCID, NOD, RAG1 or RAG2 gene mutations yielded more severe immunocompromized mice (Rag2^−/−^ cγ^−/−^ , Rag1^−/−^ cγ^−/−^ (RG), NOD/shi-scid/cγ^−/−^ null (NOG) and NOD/SCID/cγ^−/−^ (NSG) mice) which were far superior for human cell engraftment [7,15,16]. Transplantation with human hematopoietic stem cells (HSC) into these mice leads to generation of all the necessary human immune cell subsets, namely T, B, NK cells, macrophages and dendritic cells [17,18]. Levels of different cell sets vary in different mouse models, for example NK cells are produced in suboptimal levels [19], but can be increased with IL-15 treatment. Both humoral and cell mediated immune responses are seen [20]. Newer refinements currently underway include introduction of human HLA Class I and II immune system and cytokine genes to generate more robust human immune responses [15,21].

## 4. Currently Used Humanized Mouse Models

Different versions of humanized mice (Hu-Mice) currently exist, each with its own advantages and disadvantages [7]. A major distinguishing feature of new Hu-Mouse models with those of the earlier versions is their ability to support primary human immune responses. A general description describing various features and their utility for testing gene therapy approaches is detailed below and summarized in Table 1.

**Table 1:** Current Humanized Mouse Models and Preclinical Gene Therapy Studies.

Model	Method (Mouse Strain)	Advantages	Disadvantages	Gene Therapy Approaches Studied
Hu-PBL	i/p injection of human PBMC. (SCID, NOD-SCID, NSG, NOG)	Easy to produce. Immediate use. Good T cell engraftment.	Lacks multilineage hematopoiesis. Lacks primary immune response. Graft versus host disease.	CCR5 shRNA [22,23]
				tat-rev shRNA [24]
				vif/pol shRNA [25]
				antisense env [24]
				fusion inhibitor [24]
				BNAb [26]
				TCR [27]
				TRIM5α [28]
				ZFN for CCR5 [29]
				ZFN for CXCR4 [30,31]
				LEDGF/p75 [32]
Hu-HSC	Intrahepatic injection of CD34^+^ HSC into neonates. Intravenous injection of CD34+ HSC into adults. (Rag2^−/−^ yc^−/−^, NSG, NOG)	Easy to produce. Multilineage hematopoiesis. Primary humoral and cellular immune responses. IgM production. Mucosal engraftment.	Weak human HLA restriction. Weak IgG production.	CCR5 shRNA [33,34]
				gag, pol shRNA [35]
				tat-rev shRNA [35,36]
				nef shRNA [37]
				LTR shRNA [38]
				antisense env [39]
				TAR decoy [34]
				fusion inhibitor [36]
				BNAb [40]
				TRIM5α [33,34]
				ZFN for CCR5 [41]
SCID-Hu	Co-implantation of human fetal liver and thymic tissue under kidney capsule. (SCID or NOD-SCID)	Abundant T cell lymphopoiesis.	Surgery needed, labor intensive. Requires human fetal tissue. No multilineage hematopoiesis. No primary immune response. Poor peripheral T cell engraftment.	Integrase antibody [42]
				tat-rev shRNA [43]
				rev shRNA [44]
				TAR decoy [43,45]
				CCR5 ribozyme [43,45,46]
				tat-rev ribozyme rev aptamer [47]
				CCR5 intrabody [48]
				TRIM5α [28]
				Transgenic TCR [49]
				RevM10 [50]
BLT	Co-implantation of human fetal liver and thymic tissue under kidney capsule with additional i/v injection of autologous CD34+ HSC. (Rag2^−/−^ yc^−/−^, NOD-SCID, NSG)	Multilineage hematopoiesis. Primary humoral and cellular immune responses. IgM production. Presence of human thymus. Human HLA T cell restriction. Mucosal engraftment.	Surgery needed, labor intensive. Requires human fetal tissue. Weak IgG production.	CCR5 shRNA [51,52] LTR shRNA [52] transgenic TCR [53] BNAb [54]

**Hu-PBL mice:** By far the easiest model to prepare, this model is created by engraftment of human mature PBMCs by i/p route into SCID, NSG or RG mouse strains [10]. Human immune cells persist for many weeks and HIV-1 can productively infect these mice. However, the injected cells eventually decline and no notable primary immune responses could be seen due to lack of de novo multilineage human hematopoiesis. Graft versus host disease is a problem, thus complicating the interpretation of results.

**SCID-hu mice:** Surgical co-implantation of human fetal thymus and liver fragments under the SCID mouse kidney capsule generates mice harboring a functional human thymus (thy/liv organoid) [9]. There is robust thymopoiesis with generation of both CD4 and CD8 mature T cells [55]. HIV infection leads to severe depletion of both mature and immature CD4 T cells. A drawback with this model is human immune responses are lacking due to the absence of the full array of human immune cells.

**Hu-HSC mice:** These are generated by transplantation with hematopoietic stem cells (HSC) into either adult or neonatal mice. IL-2Rcγ^−/−^ mice such as RG, NOG or NSG mice are commonly utilized [7,13]. Engraftment of HSC into conditioned newborn mice via routes such as intra-hepatic injection results in superior human cell engraftment with generation of a full complement of T cells, B cells, macrophages, NK cells and dendritic cells [17,18]. HIV infection leads to chronic viremia lasting more than a year with concomitant CD4 T cell loss [56]. Human antibody responses are seen [57]. While cell mediated immune responses are detected, they are not human HLA restricted [58]. Mucosal human cell engraftment permits HIV vaginal transmission, thus permitting viral challenges by the natural route of infection to assess viral resistance conferred by anti-HIV genes [54,59,60].

**BLT mice**: These are created by a modification and improvement of the earlier SCID-hu mouse model by additional engraftment with autologous HSC (bone marrow, liver and thymus) [61,62]. Superior human cell engraftment with multilineage generation of T cells, B cells, macrophages, NK cells and dendritic cells is seen. Presence of a functional autologous human thymus in this model permits appropriate T cell education and human HLA cell restriction. HIV infection leads to viremia and helper CD4 T cell loss. Good mucosal human cell engraftment is seen thus permitting mucosal viral challenges [63]. 

## 5. Preclinical Gene Therapy Questions that Can be Effectively Assessed in Humanized Mice

*In vitro* evaluation of various anti-HIV gene therapy constructs in lab adopted T cell lines and human PBMCs provides preliminary efficacy data. However, many important physiological questions cannot be answered by these. Therefore, it is essential that promising strategies be tested *in vivo*. Among these are, how long does the efficacy last and do escape viral mutants arise after prolonged use? What types of toxicities such as adverse cytokine production are associated with *in vivo* use? For stem cell-based strategies it needs to be evaluated whether a particular anti-HIV construct displays adverse effects on the stem cell lineage specific differentiation into the end stage hematopoietic cells such as T cells, B cells, macrophages and dendritic cells. Is there any gene silencing/deletion occurring during prolonged *in vivo* application? Do some specific gene transduced cells have preferential clonal expansions and possibly have oncogenic potential? With specific gene transducing vectors it also needs to be determined if there is a preference to integrate into selective chromosomal sites. Can different anti-viral constructs when introduced in combination work synergistically in providing anti-viral protection? Finally, are the gene transduced lineage specific differentiated cells functionally competent and work synergistically with other cell types in generating an effective immune response such that immune functions can be restored in the AIDS patient? Features that distinguish the newer generation hu-HSC and BLT humanized mouse models from those of previous hu-PBL and SCID-hu mouse models are *de novo* multilineage human hematopoiesis and the capacity for generating both adaptive and innate immune responses [15,20]. Therefore many of the above questions can be effectively evaluated in an experimental setting to derive important pre-clinical data.

## 6. Anti-HIV Gene Therapy Constructs

HIV is a highly evolved complex retrovirus with many unique features, especially a great tendency for genomic variation [64]. Antigenic variation in the infected host frustrates the immune system, thus posing a difficulty in controlling infection and in designing effective vaccines. Therefore, novel intracellular immunization strategies offer innovative avenues. Whereas mature CD4 T cells can be genetically modified to confer anti-viral resistance and/or potent effector functions, targeting these approaches to HSC will be long-lasting and therefore potentially conferring a permanent viral control [5,6]. For these strategies to be effective, vulnerable steps of the viral infection need to be targeted. To date, a great deal of data has accumulated on various stages of viral replication. Steps that are amenable to preventive/therapeutic targeting encompass both cellular and virally-encoded molecules. Among these are cellular receptors CD4, CCR5 and CXCR4, virally-encoded regulatory molecules such as tat and rev, and finally host factors that assist or restrict viral replication [5,8]. Varieties of anti-HIV constructs have been experimented both *in vitro* and *in vivo*. These fall into two broad categories, namely, nucleic acid based and protein based. A particular advantage with the nucleic acid based approaches versus protein-based is that they are not immunogenic and thus are more suitable for long-range application. The following is a brief description of the various constructs and how they have been successfully evaluated in humanized mice. For brevity, only representative examples are discussed with some historical perspective.

## 7. RNAi

The phenomenon of RNA interference (RNAi) is a native cellular process that can be exploited to silence any gene of choice [65]. It involves small RNAs that can perform post-transcriptional gene silencing (PTGS) as well as transcriptional gene silencing (TGS). TGS involves the prevention of transcription through epigenetic modification of DNA sequences. This approach is beginning to be harnessed to silence HIV [66]. PTGS involves either sequence specific cleavage of fully complementary target RNAs by siRNAs or translational inhibition and destruction of mRNAs that have imperfect complementary miRNAs. These pathways involve a complex set of intracellular reactions that employ a number of cellular proteins, with the final cleavage of the target RNA performed by an RNA induced silencing complex (RISC), which acts by endonuclease activity [67]. Since the discovery of RNAi, numerous reports described use of siRNAs to inhibit HIV *in vitro*, and some of these studies progressed to *in vivo* evaluation [5,8,68]. Both cellular and viral molecules essential for viral infection were targeted. For synthetic siRNA delivery into cells, most methods used a variety of transfection methods in addition to using novel molecules such as dendrimers [69,70]. For infected cell-specific delivery, ligand-based approaches such as using antibodies or aptamers were employed [71,72,73,74]. The gene therapy approach calls for constitutive endogenous expression requiring incorporation of siRNA coding sequence into the target cell genomes. In this regard lentiviral vectors are the most commonly used for gene transduction [8].

One particular complication with the use of RNAi as a gene therapy for HIV involves the ability of HIV to generate escape mutations relatively easily. Even silent mutations with no consequences on the amino acid sequence (thus not conferring a fitness cost) could be sufficient to confer resistance to shRNA targeting viral transcripts [75]. Thus, for RNAi to be fully effective against HIV, it should be deployed as part of a combinatorial approach, either targeting multiple viral genes, or alternatively acting against cellular factors necessary for viral infection and/or replication.

Among the prominent host cell targets evaluated in the RNAi context are the primary cell surface receptor CD4 and co-receptors CXCR4 and CCR5 [6,8]. Of these CCR5 received the most attention recently due to the now famous case of the “Berlin patient” who was cured of the HIV infection after allogenic bone marrow transplantation with CCR5 negative HSC [76,77]. Based on this example, many ongoing studies are directed towards silencing the CCR5 gene.

In early studies evaluating CCR5 siRNAs using gene transduced human PBMCs *in vivo* in humanized mice, An *et al.* found that high expression levels achieved under the control of the U6 promoter resulted in significant cytotoxicity [78]. However, this was avoided through the use of a weaker H1 promoter but resulting in the desirable levels of CCR5 down regulation. In later studies using a similar approach, CD34 HSCs were transduced and engrafted into BLT mice [51]. T cell development was normal and CCR5 knockdown was maintained during long term *in vivo* and these cells were resistant to HIV challenge *ex vivo.* For targeted delivery of CCR5 shRNA only into CCR5-expressing cells, a ZZ domain/monoclonal antibody conjugated Sindbis virus glycoprotein pseudotyped lentiviral vector was used [23]. *In vitro* results showed inhibition of HIV-1 replication, and *in vivo* results with NSG mice engrafted with these cells confirmed CCR5 expressing cells were targeted for transduction. Transduction of iPSCs with a lentiviral vector encoding CCR5 shRNA has also been tested, however, the *in vivo* efficacy of the transduced cells was not demonstrated [33]. A potential limitation of targeting only CCR5, however, is that such a strategy may promote selection of a CXCR4-tropic strain, with the possibility of a quicker progression to AIDS.

In addition to host cell molecules, a number of virally-encoded RNAs were also targeted by siRNAs. Some examples are, *tat*, *rev*, *nef* and e*nv*. Many of these were successfully tested in humanized mice (See Table 1). SiRNAs against the viral tat-rev genes were found to be effective at inhibiting HIV *in vivo* and *ex vivo* [24,36,43]. shRNA for *rev* alone [44], *nef* alone [37], *vif/pol* [25], and the HIV LTR [38,52] have been shown to have efficacy as well in humanized mice, along with the full length antisense env gene [24,39]. More recently the safety of a multi-shRNA based gene therapy for HIV-1 targeting gag, pol and tat/rev, has been evaluated in humanized mice [35]. Phase I clinical trials using this construct are planned.

## 8. Ribozymes

Ribozymes are RNA molecules with catalytic activity which can cleave phosphodiester bonds present in target RNA sequences, and thus can be exploited for gene silencing. They can be custom designed to achieve site specific cleavage of desired target RNA molecules [78]. There was much initial excitement in using ribozymes for clinical application, although later discovery of RNAi provided more potent molecules for similar applications. For HIV gene therapy, ribozymes were employed to target either host or virally-encoded RNAs, some of which were later evaluated in clinical trials [79,80]. Modest decreases in viral load and increases in frequencies of CD4 T cells were seen in treated individuals [80]. Similar to siRNAs, ribozymes are also non-immunogenic, thus avoiding the risk of immune rejection of gene transduced cells.

In early *in vivo* studies of CCR5, specific ribozymes were incorporated into retroviral vectors and transduced into HSCs for gene therapy application [6]. Vector delivered ribozymes have played a key role in setting the stage for deriving preclinical data on transgene effects on hematopoietic lineage specific differentiation and expression in terminally differentiated cells such as T cells [42]. In these studies, the SCID-hu mouse model was used. Work of Bai *et al.* that tested a CCR5 ribozyme showed no adverse effects on thymocyte differentiation from retroviral gene transduced HSC [46]. Subsequent studies using a lentiviral vector that gave much higher transduction efficiencies showed similar results [45]. *In vivo* derived transgenic cells were found to resist *ex vivo* HIV challenge confirming the efficacy of the ribozyme in down regulating the CCR5 co-receptor in differentiated cells. Later studies in SCID-hu mice have also successfully tested the efficacy of anti-HIV *tat-rev* and *env* ribozymes [47]. In a combinatorial approach, CCR5 ribozymes were incorporated into a lentiviral vector that also harbored a TAR decoy and a tat-rev siRNA and tested in humanized mice [43] (see below).

## 9. RNA Decoys and Aptamers

RNA decoys consist of the protein binding sequences of parent native RNA molecules whereas RNA aptamers are derived by *in vitro* selection for high affinity binding [69]. These molecules sequester the cognate viral regulatory or essential proteins and neutralize their function. TAR decoys inhibit HIV by neutralizing viral tat protein and rev aptamers interfere with rev function. Both TAR decoys [45] and a rev aptamer [47] were evaluated in SCID-hu mice. Transgenic T cells derived *in vivo* were found to show anti-HIV resistance. Later studies used the TAR decoy in combination with anti-CCR5 ribozymes and tat-rev siRNAs [43], and a TAR decoy in combination with CCR5 shRNA and a chimeric human-rhesus macaque TRIM5α gene was shown in hu-HSC mice to have *in vivo* efficacy against HIV [34]. Many other aptamers to different viral or cellular targets were developed for use in gene therapy approaches and await *in vivo* testing in a gene therapy setting [81]. With regard to immune rejection, RNA decoys and aptamers are also considered to be safe due to lack of immunogenicity.

## 10. Antibodies

While antibody responses are typically generated in response to HIV-1 infection, they are found to be inadequate in control of viral replication due to the propensity of the virus for constant antigenic variation [82]. This feature of the virus has also been frustrating in the development of an effective vaccine. Therefore, novel gene therapy efforts have also been directed at boosting host humoral immunity by delivering anti-HIV antibody coding genes into human cells *in vivo.* A first report utilizing this strategy employed an SV40 vector encoding a variable antibody fragment against the viral integrase protein [42]. SCID-hu mice thy/liv grafts were injected with the vector leading to the production of antibody by the transduced thymocytes. HIV challenge in mice showed marked viral resistance, supporting the value of this approach. Broadly neutralizing anti-HIV antibodies (bNAbs) are seen in a subset of HIV infected individuals [82]. These bNAbs are superior due to their high potency against a broad range of HIV strains, and therefore vaccines that can elicit these will have obvious advantages. However, induction of these in immunized individuals has not been accomplished with the current experimental vaccines. Therefore, supplementation of these via what is recently termed as vectored immunoprophylaxis shows considerable promise.

Such an approach was first tested by using bNAb 2G12 in humanized mice [40]. An antibody encoding lentiviral vector was employed to transduce human HSC, which were then engrafted into NSG mice. Systemic 2G12 antibody production was seen, and there was marked reduction in viral loads upon HIV challenge compared to non-treated mice. A separate approach used a tumor cell “backpack” constitutively producing a dimeric 2G12 bNAb. Hu-Mice harboring this “backpack” showed reduced viral loads and preserved CD4 T cell levels [83]. Using a different vector, AAV coding for bNABs b12, 2G12, 4E10 and VRC01, humanized mice (NSG and RG mice backgrounds) were vaccinated intramuscularly, leading to systemic antibody production lasting as long as 52 weeks [84]. Full protection from HIV challenge via i/v route was seen with the B12 and VRC01 antibodies, whereas partial protection was seen with 2G12, 4E10, and 2F5 antibodies. Since HIV is transmitted primarily by sexual transmission by the mucosal routes and IgA is the predominant mucosal antibody, another study evaluated the efficacy of lentiviral vector delivered IgA [54]. In humanized NSG, BLT, and hu-HSC RG mice prepared by transplantation with antibody gene transduced HSC, the transgenes were expressed by B cells and plasma cells in both lymphoid organs and mucosal sites. While there was no protection from HIV infection following HIV-1 vaginal challenge, CD4 T cell depletion was drastically reduced in mucosal sites.

Antibodies directed against host proteins involved in viral infection have also been tested in humanized mice in a different approach. CD34^+^ HSCs were transduced with a lentiviral vector encoding a single chain CCR5 antibody (termed intrabody). During intracellular synthesis the intrabody prevents export of CCR5 protein to the cell surface. SCID-hu mice engrafted with the intrabody transduced HSC were found to be resistant to R5 tropic HIV infection [48].

## 11. Transgenic T cell Receptors

Well chosen viral specific T cell receptors (TCRs) genetically introduced into primary T cells or HSCs have the potential to mediate potent effector T cell functions against infected cells expressing conserved viral antigens [8]. Therefore their exploitation for HIV gene therapy holds considerable promise. *In vitro* studies have documented the efficacy of such gene transduced CD8 T cells bearing transgenic TCRs in inhibiting HIV replication [84]. *In vivo* efficacy was evaluated by the use of a lentiviral vector containing a gag-SL9 (SLYNTVATL) specific TCR. Vector transduced human PBMC were injected into the spleens of SCID mice along with HIV infected PBMC [27]. Significantly lower viral titers were seen, suggesting that the engineered CTLs were able to exert some control over viral replication *in vivo*. In a stem cell based approach, human HSC transduced with a similar gag-SL9 TCR and allowed to mature in SCID-hu mice thy/liv grafts [49]. Functional HIV specific CTLs were generated which recognized SL9 epitope in the context of proper HLA type. Later, this same group demonstrated *in vivo* suppression of HIV in NSG BLT mice prepared by injection of HSC transduced with gag-SL9 TCR [53]. Higher numbers of SL9-specific CD8 T cells *in vivo* correlated with lower plasma viral loads. These data demonstrated the feasibility of cell mediated immune engineering against HIV. However, for a broad and long lasting protection it is necessary that multiple TCRs must be chosen carefully to target the most conserved HIV epitopes, since escape from CTL responses is common [85].

## 12. Transdominant Proteins

Transdominant proteins exert their effect by interfering with the action of their counterpart native proteins involved in critical functions essential for viral replication. Some of these with anti-HIV properties have been evaluated in humanized mice to determine protection from HIV-1. An example is RevM10 that interferes with HIV rev protein action [50]. RevM10 encoding retroviral vector transduced CD34 HSC when engrafted into SCID-hu thymic grafts gave rise to normal T cells which upon *ex vivo* challenge showed HIV resistance. However, a disadvantage in a clinical setting is the immunogenicity of the protein leading to eventual elimination of gene transduced cells *in vivo*. Indeed in clinical trials this has not fared well, resulting in only a modest survival of CD4 T cells containing the RevM10 gene [86,87,88].

Another target of HIV-1 gene therapy tested in humanized mice is Lens Epithelium Derived Growth Factor (LEDGF) which is an essential cellular cofactor for viral integration [89]. Its inactivation by siRNAs or over expression of its C-terminal domain was shown to inhibit HIV-1 *in vitro*. This effect was also confirmed *in vivo* in humanized mice using gene transduced CD4 T cells [32]. Lower viral loads and protection from CD4 T cell decline were observed.

## 13. Zinc finger, TALENS and CRISPR Nucleases

Novel gene disruption methods employ gene targeted nucleases which encompass ZFN (zinc finger nucleases), TALENS (transcription activator-like effector nucleases), and CRISPR (clustered regulatory interspaced short palindromic repeats) systems [90,91,92,93]. ZFNs were among the nucleases initially exploited to silence the CCR5 gene to render HIV susceptible cells impervious to infection with the R5 tropic HIV, the predominant viral strain involved in natural viral transmission. Engineered ZFNs, which are an array of DNA-binding zinc fingers tethered covalently to a nonspecific FokI restriction nuclease, act by causing double strand DNA breaks at specific recognition sites leading to gene deletions and insertions thus permanently disabling a targeted gene [94]. An advantage with these is that their transient expression can lead to permanent gene disruption. In some of the first studies with CCR5 specific ZFNs, Perez *et al.* showed that human CD4 T cells that had been modified by ZFNs showed efficacy in humanized mice [95], with lower viral loads and preserved CD4 T cell levels. Later studies of Holt *et al.* also evaluated this approach *in vivo* in humanized mice using ZFN modified HSCs [41]. There was multilineage human hematopoiesis and generation of cells lacking the CCR5 receptor. HIV infection of these mice resulted in selection of CCR5 negative cells, lower viral loads and protection from CD4 T cell loss. More recently, an adenoviral vector encoding CCR5-ZFNs was used to modify human CD4 T cells, which were then engrafted into NSG mice to evaluate potential toxicity [29]. No detectable ZFN-specific toxicity or T-cell transformation was observed.

ZFNs targeting the second coreceptor CXCR4 have also been evaluated in humanized mice [30,31]. When engrafted with zinc-finger modified CD4 T cells and challenged with X4 tropic virus, these mice were shown to be resistant to *in vivo* challenge. However, disabling CXCR4 in HSCs has disadvantages since this receptor is indispensable for normal homing patterns and hematopoietic homeostasis. Potential downsides to the nuclease-based gene disruption approaches include the relatively modest bi-allelic gene modification and off target effects [96], resulting in unwanted disruption of important regulatory genes, possibly leading to malignancy. While no short term toxicities have been observed so far in ongoing clinical trials [29], long term follow up for several years is required to assess potential negative effects.

TALENs, like ZFNs, bind to specific DNA sequences via transcription activator-like (TAL) proteins originally isolated from plant-pathogenic bacteria of the *Xanthomonas* species [97]. They create double stranded breaks through the action of covalently bound FokI nuclease domain [94]. They have an advantage of higher sequence specificity than ZFNs and relatively simple retargeting, with the disadvantage of a larger size and subsequent greater difficulty in delivery to target cells.

Originally identified as elements of an adaptive immune system found in bacteria and archea, CRISPR, in constrast to ZFN and TALENs, relies on the use of guide RNA (gRNA) to deliver CRISPR-associated (Cas) nucleases [98]. When the gRNA bound to a codon-optimized Cas9 protein binds to a complementary sequence on DNA, it creates double strand DNA breaks at the unique target sequences. This has the advantages of even greater sequence specificity and is relatively easy to retarget to new sequences [99]. Potential off-target effects remain to be evaluated in future in depth studies.

There has been considerable excitement in the use of TALENs or CRISPR to target the latent viral reservoir through disruption of the proviral genome. One *in vitro* study involving the CRISPR/Cas9 system demonstrated inhibition of an HIV LTR [100]. *In vivo* studies on the use of TALENs and CRISPR for HIV prevention and therapies are the next step to establish their future clinical application.

## 14. Host Restriction Factors

As a host defense, mammalian cells encode many dominant acting proteins that help suppress viral replication. These are termed host restriction factors [101]. With regard to HIV restriction, a number of such factors were identified. Prominent ones are TRIM5α, TRIMcyp, APOBEC 3F and G, SAMHD1 and tetherin, which can potentially be harnessed for HIV gene therapy. Many *in vitro* studies evaluated their anti-HIV effects. With regard to *in vivo* testing in humanized mice, early reports used a chimeric human-rhesus TRIM5α [28], which was humanized to reduce potential immunogenicity while retaining the critical rhesus macaque motif responsible for HIV restriction. HSCs were transduced with a lentiviral vector containing this construct and introduced into SCID-hu grafts. The transgenic T cells matured in these mice were found to be HIV resistant. In a different study, a human version of the monkey AoTRIM5Cyp (hT5Cyp) was transduced into human CD4 T cells and evaluated in hu-PBL mice [102]. Viral challenge experiments demonstrated decreased HIV viral loads and protection from CD4 T cell decline. Chimeric versions of TRIM5α in combination with other anti-HIV genes were also tested *in vivo* in humanized mice with encouraging results [34] (see below). A note of caution with the use of modified restriction factors for long-range gene therapy, however, is the potential for generating unwanted human immune responses, thus mitigating the expansion and survival of these transgenic cells *in vivo*. 

## 15. Fusion Inhibitors

Clinically licensed peptide drugs such as enfuvirtide (T20) inhibit HIV entry by interfering with viral fusion with the cell membranes [103]. A fusion inhibitor C46 (derived from a 46 amino acid sequence of gp41) with a similar structure to T20 can be stably expressed as a membrane anchored peptide (maC46) in retro or lentiviral vector transduced cells thus enabling its use as a gene therapeutic construct. C46 was tested in hu-PBL mice by introducing gene transduced CD4 T cells which showed efficacy [24]. When compared with two other anti-HIV constructs, namely a tat-rev siRNA and an antisense RNA VRX494, it was found to confer higher selective survival advantage *in vivo*. These studies did not address potential immunogenicity of the peptide, however, which remains a concern for long term efficacy.

## 16. Combinatorial Approaches

Due to the ability of HIV to rapidly mutate and escape under selective pressure, the early mono-antiretroviral therapies were not fully effective during long-term use. Use of combinatorial ART (HAART) has overcome this problem to a large extent [1]. Similarly, use of a single anti-HIV gene therapeutic construct will invariably lead to generation of viral escape mutants thus requiring the design and utilization of an ideal combinatorial construct for effective viral control. Based on this premise, many studies investigated this approach using viral vectors that harbor multiple genes [5]. Ideally, a combination of anti-HIV constructs with different mechanisms of action targeted to different stages of viral life cycle is desirable. A number of different constructs that include ribozymes and siRNAs targeted to both cellular and viral RNA targets, fusion proteins and restriction factors in different combinations were evaluated, some of which have been tested *in vivo* in humanized mice. Among these, one study employed a triple anti-HIV gene encoding lentiviral vector harboring a TAR decoy, CCR5 ribozyme and a tat-rev siRNA [43]. HSCs transduced with this combinatorial vector gave rise to HIV resistant cells when engrafted into SCID-hu mouse thy/liv grafts, providing data for a subsequent human clinical trial. In another study, a triple combination of CCR5 siRNA, chimeric human/rhesus TRIM5a and TAR decoy was evaluated in hu-HSC mice [34]. These studies, in addition to showing anti-viral efficacy, also demonstrated no apparent toxicity with the combination of genes, although neither study directly compared the effectiveness of combination therapy versus their respective single therapies. An *in vitro* study, however, showed that while HIV-1 can escape from a single shRNA, this was not the case when four anti-HIV shRNAs were expressed in the same cell [104]. As can be seen, humanized mice have been instrumental for deriving important pre-clinical data.

## 17. Clinical Studies and Future Directions

While numerous *in vitro* and *in vivo* studies laid the groundwork by identifying a large variety of anti-HIV constructs for testing gene therapy strategies, only a few of these reached clinical trials [105] (summarized in Table 2). Most were phase I studies aimed at safety and feasibility. Previous stem cell based trials involved retroviral vectors harboring RevM10 transdominant protein [88], RRE decoy [106,107] or anti-HIV ribozymes [80,108,109,110]. No adverse effects were seen in patients receiving the treatment, thus showing safety. While there were detectable levels of transgene expressing cells, the gene marking levels were too low, however, to provide any durable clinical benefit. In a recent combinatorial approach using HSC, the triple lentiviral vector described above containing a CCR5 ribozyme, TAR decoy and a tat-rev siRNA was tested in AIDS lymphoma patients [79]. Again, levels of gene marking were low and there was no clinical benefit. Many phase I/II clinical trials also evaluated gene transduced T cells, again showing less than ideal levels of gene marking and persistence of gene modified cells [105]. Therefore, it is clear that a number of important hurdles need to be overcome to bring HIV gene therapy to a clinical reality. Among the main ones are “making space” for gene transduced HSC by myeloablation methods to allow durable engraftment, protocols to enable selection of gene modified cells, and high level gene transduction of the true and long lasting subpopulation of HSCs. In this regard, humanized mice can be exploited to evaluate new innovative experimental strategies to realize the full potential of gene therapy approaches in a clinical setting. 

**Table 2:** HIV Gene Therapy Clinical Trials.

Gene therapy construct (viral or cellular target)	Proprietary name	Gene modified cells	Delivery method	Phase, status	Reference(s)
Antisense (env mRNA)	VRX496	Autologous CD4^+^ T cells	Lentiviral vector	I-II, Ongoing	[111,112,113]
					*NCT00622232
					*NCT00295477
					*NCT00131560
ZFN (CCR5 gene)	SB 728T	Autologous CD4^+^ T cells	Adenoviral vector	I-II, Ongoing	[114]
					*NCT01543152
					*NCT01252641
					*NCT01044654
					*NCT00842634
shRNA (CCR5 mRNA) Fusion inhibitor C46 (env protein)	Cal-1	Autologous CD34+ HSCs and CD4+ T cells	Lentiviral vector	I-II, Ongoing	[115]
					*NCT01734850
Fusion inhibitor C46 (env protein)	M87o	Autologous of Allogeneic CD34^+^ HSCs	Retroviral vector	I-II, Ongoing	[116]
					*NCT00858793
Endoribonuclease (ACA sequences)	MazF-T	Autologous CD4^+^ T cells	Retroviral vector	I, Ongoing	*NCT01787994
Transgenic TCR (gag epitope)		Autologous CD8^+^ T cells	Lentiviral vector	I, Ongoing	*NCT0091224
Chimeric antigen receptor (gp120 protein)		Autologous CD4^+^ and CD8^+^ T cells	Retroviral vector	I-II, Completed	[117,118,119,120]
					*NCT00001409
					*NCT01013415
Antisense (TAR, tat/rev mRNA)	HGTV43	Autologous CD34^+^ HSCs	Retroviral vector	I-II, Ongoing	[121]
Ribozyme (tat/vpr mRNA)	OZ1	Autologous CD34^+^ HSCs	Retroviral vector	II, Ongoing	[80,122]
					*NCT01177059
					*NCT00074997
Ribozyme (tat/vpr mRNA)	Rz2	Syngeneic CD4^+^ T cells	Retroviral vector	I, Completed	[108,123,124,125]
Ribozyme (tat/rev mRNA)		Autologous CD34^+^ HSCs	Retroviral vector	II, Completed	*NCT00002221
shRNA (tat/rev mRNA) TAR decoy (tat protein) Ribozyme (CCR5 mRNA)		Autologous CD34^+^ HSCs	Lentiviral vector	Pilot, Ongoing	[79]
					*NCT01153464
					*NCT00569985
Ribozyme (U5/pol mRNA)	MY-2	Autologous CD4^+^ T cells	Retroviral vector	I, Completed	[110]
RRE decoy (rev protein)		Autologous CD34^+^ HSCs	Retroviral vector	Pilot, Completed	[107]
Transdominant rev (rev protein)		Autologous CD34+ HSCs	Retroviral vector	I, Completed	[126,127]
Transdominant rev (rev protein)		Autologous CD4^+^ T cells	Gold particles	I, Completed	[128]
Transdominant rev (rev protein)		Autologous CD4^+^ T cells	Retroviral vector	I, Completed	[88]
Transdominant rev (rev protein) Antisense (pol mRNA)		Autologous CD34+ HSCs	Retroviral vector	I/II, Completed	*NCT00003942

* clinicaltrials.gov reference number
